# Genetic and ecological insights into glacial refugia of walnut (*Juglans regia* L.)

**DOI:** 10.1371/journal.pone.0185974

**Published:** 2017-10-12

**Authors:** Mallikarjuna Aradhya, Dianne Velasco, Zakir Ibrahimov, Biimyrza Toktoraliev, David Maghradze, Mirza Musayev, Zviadi Bobokashvili, John E. Preece

**Affiliations:** 1 National Clonal Germplasm Repository, USDA-ARS, University of California Davis, Davis, California, United States of America; 2 Department of Plant Sciences, University of California Davis, Davis, California, United States of America; 3 Department of Environmental Engineering and Forestry, Azerbaijan State Agricultural University, Ganja, Azerbaijan; 4 National Academy of Sciences of the Kyrgyz Republic, Bishkek, Kyrgyz Republic; 5 Institute of Horticulture, Viticulture, and Oenology, Agricultural University of Georgia, Tbilisi, Georgia; 6 Laboratory of Subtropical Plants and Grapevine, Genetic Resources Institute, Baku, Azerbaijan; 7 Scientific Research Center of Agriculture, Ministry of Agriculture, Tbilisi, Georgia; University of Colorado, UNITED STATES

## Abstract

The distribution and survival of trees during the last glacial maximum (LGM) has been of interest to paleoecologists, biogeographers, and geneticists. Ecological niche models that associate species occurrence and abundance with climatic variables are widely used to gain ecological and evolutionary insights and to predict species distributions over space and time. The present study deals with the glacial history of walnut to address questions related to past distributions through genetic analysis and ecological modeling of the present, LGM and Last Interglacial (LIG) periods. A maximum entropy method was used to project the current walnut distribution model on to the LGM (21–18 kyr BP) and LIG (130–116 kyr BP) climatic conditions. Model tuning identified the walnut data set filtered at 10 km spatial resolution as the best for modeling the current distribution and to hindcast past (LGM and LIG) distributions of walnut. The current distribution model predicted southern Caucasus, parts of West and Central Asia extending into South Asia encompassing northern Afghanistan, Pakistan, northwestern Himalayan region, and southwestern Tibet, as the favorable climatic niche matching the modern distribution of walnut. The hindcast of distributions suggested the occurrence of walnut during LGM was somewhat limited to southern latitudes from southern Caucasus, Central and South Asian regions extending into southwestern Tibet, northeastern India, Himalayan region of Sikkim and Bhutan, and southeastern China. Both CCSM and MIROC projections overlapped, except that MIROC projected a significant presence of walnut in the Balkan Peninsula during the LGM. In contrast, genetic analysis of the current walnut distribution suggested a much narrower area in northern Pakistan and the surrounding areas of Afghanistan, northwestern India, and southern Tajikistan as a plausible hotspot of diversity where walnut may have survived glaciations. Overall, the findings suggest that walnut perhaps survived the last glaciations in several refugia across a wide geographic area between 30° and 45° North latitude. However, humans probably played a significant role in the recent history and modern distribution of walnut.

## Introduction

Paleobotanical studies suggest Quaternary climatic fluctuations beginning in Plio-Pleistocene transition profoundly impacted biodiversity and altered the floristic composition throughout the Holarctic [1–4]. Further, recurrent oscillations between glacial and interglacial periods during the Pleistocene caused massive extinction of species in the European tree flora [5, 6]. Temperate tree diversity began to decline with the onset of glaciations in the Late Pliocene extending into the Middle Pleistocene and majority of the Pliocene temperate trees did not survive to the present [5–8]. Nonetheless, paleobotanical evidence indicate that some did survive in isolated refugia during the last glacial maximum (LGM), both above and below glacial boundaries [9–11]. During the LGM nemoral trees that are generally associated with broad-leaved forests were confined to the southern Mediterranean, Black, and Caspian Sea regions [12, 13]. Temperate trees that survived in northern cryptic refugia [10, 11] experienced a series of bottlenecks, rapidly losing genetic diversity with interglacial expansions and contractions, leading to the disappearance of cryptic refugia [5, 8]. Present day temperate trees in Eastern Europe are therefore the result of range expansion from southern refugia following the retreat of ice sheets. Pleistocene refugia have traditionally been identified based on paleobotanical and historical biogeographic evidence. Recently, population genetic studies in conjunction with paleoreconstruction of species distributions have offered insights into genetic consequences of glacial episodes [2, 14–16].

English walnut (*Juglans regia* L.; henceforth referred to as walnut) belongs to the section *Juglans* within the genus *Juglans* of the family Juglandaceae and is considered to be a Neogene relict from the Tertiary forests of Eurasia [17–21]. Walnut has been documented in Eurasia from the middle of Paleogene through Neogene and later in the Mediterranean region during the Pliocene transgression [22–24]. The evolutionary history of the section *Juglans* is riddled with widespread extinctions, range reduction, fragmentation, and bottlenecks during the Late Tertiary climatic deterioration and Quaternary glaciations. Palynological data indicate that walnut populations were extirpated from Eastern Europe to southwestern Turkey at the end of the LGM [25–28]. However, small isolated populations of walnut probably survived in glacial refugia in the Mediterranean, the Black Sea (Euxinian vegetation), and the Caspian Sea (Hyrcanian vegetation) regions as far east as the Balkans and up north in the Carpathian region [19, 21, 26, 29] and west into southern Italy [28, 30] and the Iberian peninsula [31]. Postglacial expansions from different refugia into higher latitudes probably occurred during the Holocene. Further human intervention played a major role in the recent history and present range expansion of walnut beyond its natural boundaries [28, 32, 33]. However, walnut from the eastern Himalayas, upper Burma, and southeastern China represent a center of diversity within the Tertiary flora of East Asia [18]. Xi [34] claimed a Chinese center of origin of walnut based on several lines of evidence; presence of a fossil species described as *J. shanwangensis* from Linju, Shanwang, Shandong provinces that resembles the modern walnut, carbonized shells found in the ruins of Cishan, Hebei, and pollen dating back to 4000–5000 BCE. But most observers believe that walnut was introduced from the Persian Empire and southern Tibet by traders along the ancient silk routes during the Han Dynasty (206 BCE-220 CE) [35].

Although the origin of walnut is obscure, it is believed to have multiple centers of origin in the Carpathian Mountains, Transcaucasia, northeastern Turkey, northern Iran, the western Tien Shan Mountains, eastern Himalayas, and the Tibetan Plateau, where a primitive endemic walnut, *J. sigillata*, exists. However, Zohary et al. [36] proposed northeastern Turkey and the southern Caucasus as the plausible centers of walnut domestication with postglacial wild walnut in the Balkans and Central Europe representing feral derivatives introduced by humans as recently as the Bronze Age. Zeven and Zhukovsky [37] considered Central Asia and adjacent Near Eastern regions as the origin and primary center of diversity of walnut. The modern day walnut represents postglacial expansion, colonization, and cultivation comprising diversity resulting from complex interactions of natural and human selection and domestication [38, 39]. Dode [40] described six taxa to accommodate the variation and ecotypic differentiation within the Eurasian populations of walnut, with additional taxa recognized by Soviet and other botanists.

Chloroplast genomic diversity has been extensively used to analyze the historical phylobiogeography of plants at interspecific and intergeneric levels, but limited organelle DNA polymorphisms make it unsuitable to study infraspecific genetic diversity, population structure, and differentiation. Alternatively, genomic DNA polymorphisms offer excellent opportunities to study spacio-temporal genetic diversity, population structure, and differentiation resulting from the dynamic interaction of evolutionary forces at infraspecific levels. This study focuses on: (1) examining the genetic structure and differentiation of modern walnut to identify the plausible hotspots of diversity, and (2) ecological niche modeling (ENM) to elucidate present and project past distributions during the last glacial maximum (LGM; 21–18 kyr BP), and the Last Interglacial (LIG or Eemian; 130–116 kyr BP). We address the following questions: (1) where did walnut survive during the LGM and LIG? (2) does the modern genetic structure and differentiation patterns provide evidence for the potential location(s) of Pleistocene refugia; and (3) does ecological niche modeling identify location(s) of refugia congruent with genetic evidence?

## Materials and methods

### Plant material, DNA extraction, and microsatellite analysis

The study used 643 genotypes comprising 317 diverse accessions representing the modern range of distribution of walnut maintained at the National Clonal Germplasm Repository, USDA-ARS, Davis, California (S1 Table). Five major distribution centers (Caucasus, Central Asia, East Asia, Southwest (SW) Asia, and Eastern Europe) were considered.

Fresh leaf tissue was collected from each accession and total DNA isolated following a standard CTAB protocol [41] and treatment with RNase A and diluted to approximately 50ng/*μ*L. Nineteen microsatellite loci, WGA001, WGA004, WGA009, WGA069, WGA089, WGA106, WGA118, WGA178, WGA202, WGA223, WGA225, WGA237, WGA318, WGA321, WGA331, WGA332, WGA338, WGA349, and WGA384 [42, 43] were amplified by polymerase chain reaction (PCR) with fluorescent labeled forward and unlabeled reverse primers. The microsatellite loci were amplified in a triplex format in a 15 *μ*L reaction mixture containing 10 mM Tris–HCl, pH 8.3, and 50 mM KCl (all included in 10X PCR buffer), 2 mM MgCl_2_, 0.9 pmol of each primer, 0.2 mM of each dNTP, 0.6 U of Taq polymerase (New England BioLabs, Ipswich, MA), and approximately 25 ng of template DNA. The PCR conditions were as follows: 1 cycle of 94°C for 5 min, 30 cycles of 94°C for 30 sec, 55°C for 30 sec, and 72°C for 40 sec, and a final elongation of 72°C for 7 min. Amplified products were resolved by capillary electrophoresis using an ABI 3130xl Genetic Analyzer with Data Collection software, version 3.0 (Applied Biosystems, Foster City, CA). The data was further analyzed using Genotyper, Version 2.5 (Applied Biosystems) and data assembled as bi-allelic genotypes (S2 Table) and in a binary matrix (1 = presence, 0 = absence) format.

### Population structure analysis

Genetic relationship among accessions was assessed by a cluster analysis (CA) using the Neighbor-Joining (NJ) algorithm as implemented in the MEGA 6.0 software [44] using a distance matrix assembled based on the proportion of alleles shared between two accession for all possible pair-wise combinations [45]. The bootstrap interior branch test [46] was used to test reliability of interior braches on the tree. The principal components analysis (PCA) was performed on the multilocus genotype data using the R package *adegenet* [47]. The accessions were projected onto a two dimensional space bound by the first two principal axes to elucidate the genetic relationships within and among geographic groups.

The genotypic data were subjected to a Bayesian model-based CA using the software package STRUCTURE 2.3.1 [48] to determine the optimum number of groups reflecting the genetic structure. STRUCURE allocates individuals into clusters (*K*) based on multilocus genotype data, so as to minimize deviations from Hardy-Weinberg and linkage equilibrium. The program uses a Markov Chain Monte Carlo (MCMC) procedure to estimate *P*(*X*|*K*), the posterior probability that the data (*X*) fit the hypothesis of *K* clusters. The analysis assigns individuals into each of the *K* clusters based on the membership coefficient (*Q*-value) which sums to unity over the number of clusters (*K*) assumed. STRUCTURE was set to ignore population information, and to use an admixture model with correlated allele frequencies, as it is considered the best option for subtle population structure [49]. The degree of admixture (*α*) was allowed to be inferred from the data. *α* is close to zero when most individuals are from one population or another, while it is greater than one when most individuals are admixed [49]. The allele frequency parameter (*λ*) was set to one as suggested in the STRUCTURE manual. From a pilot study, we found that burn-in and MCMC simulation lengths of 100,000 replicate runs were optimum to achieve accurate parameter estimates. We let the number of clusters (*K*) vary between 2 and 18 with 20 replicate runs to quantify the variation of the likelihood for each *K*. The *K* value that provides the maximum likelihood (Ln *P*(*D*) in STRUCTURE) across runs is generally inferred as the most probable number of clusters. However, the interpretation of *K* should be treated with care as it merely provides an *ad hoc* approximation [48] and sometimes genuine and subtle population structure may be missed by STRUCTURE. Therefore we used an *ad hoc* statistic Δ*K* to choose the optimum number of clusters (*K*) based on the second order rate of change in the log probability of data between successive *K* values as proposed by Evanno et al. [50].

### Genetic diversity within and among groups

The multilocus genotype data were pooled into five geographic groups matching the results of the CA and subjected to analysis of total and within-group genetic diversity measures such as mean number of alleles per locus (*A*), observed (*H*_*o*_) and expected (*H*_*e*_) levels of heterozygosity, and fixation index (*F*) for different loci. Allelic richness (*Ar*) and private allelic richness (*PAr*) for each population were estimated using the rarefaction method [51], which compensates for differences in sample size (i.e. rarified allelic richness) among populations as implemented in hp-rare 1.1 [52]. The estimates of *Ar* and *PAr* were geographically projected using an inverse distance weighted (IDW) interpolation tool implemented in the ArcMap 10.1 (ESRI, Redlands, CA USA). Gene diversity analysis was performed on the allele frequency data from the five geographic groups by following the method suggested by Nei [53]. The total gene diversity (*H*_*T*_) was partitioned into gene diversity due to variation within groups (*H*_*G*_), and the component due to variation between groups (*D*_*GT*_). Differentiation between groups was calculated as *G*_*GT*_ = *D*_*GT*_/*H*_*T*_, where *G*_*GT*_ can vary between 0 (when *H*_*G*_ = *H*_*T*_) and 1 (when *H*_*G*_ = 0), i.e. groups fixed for different alleles.

The group-wise microsatellite data were also analyzed using the analysis of molecular variance (AMOVA) as implemented in the software package ARLEQUIN version 3.6 [54]. The total variance was partitioned into variation within and among groups. The variance components from AMOVA were used to estimate the population subdivisions within and among groups. Contingency *χ*^2^ analysis was performed to determine the heterogeneity among groups before performing AMOVA. A population pair-wise *F*_*ST*_ matrix was computed to assess genetic differentiation among different geographic groups.

### Ecological niche modeling

We used 237 unique walnut occurrence locations with corresponding georeferenced data gleaned from the Genetic Resources Information Network (GRIN, USDA-ARS; http://www.ars-grin.gov/npgs/index.html), the Global Biodiversity Information Facility (GBIF; http://www.gbif.org), field collections, and published literature (S3 Table) representing the current walnut distribution. Modeling of modern distribution of walnut was performed using the maximum entropy algorithm implemented in MaxEnt 3.3.3e [55] with the current climatic data from the WorldClim database [56]. Past climatic data from two general circulation models (GCM), the Community Climate System Model (CCSM) [57], and the Model for Interdisciplinary Research on Climate (MIROC, version 3.2; [58]) at 2.5’ spatial resolution, were used to hindcast LGM distributions. Data for LIG [59] at 0.5’ spatial resolution aggregated to 2.5’ resolution were used to model LIG distribution. Highly correlated environmental variables (Pearson’s correlation coefficient >0.7) were excluded from modeling, leaving eight bioclimatic variables: mean annual temperature, mean diurnal range, isothermality, temperature seasonality, mean temperatures of the wettest quarter, mean temperature of the driest quarter, annual precipitation, and precipitation seasonality.

#### Correction of sampling bias

The occurrence data often exhibit spatial autocorrelation and could potentially introduce environmental bias into modeling [60–63]. In order to minimize environmental bias, we filtered walnut data using the rarefying tool in the species distribution model (SDM) toolbox [64] implemented in ArcMap 10.1 (ESRI, Redlands, CA). We rarefied data at 10 and 25 km spatial resolutions based on climatic heterogeneity of the mountainous regions where the samples originated. The filtering resulted in 136 and 112 unique localities for the 10 and 25 km rarefying resolutions, respectively.

Presence-only data are inherently biased due to uneven sampling over the species landscape [65]. In order to infer meaningful information from such data we need to correct for the sampling bias. We account for sampling bias by providing MaxEnt with a bias grid of the sampling probability surface roughly representing the sampling efforts and giving weights to random background data used for modeling. Ideally a bias file would represent the actual sampling intensity across a large rectilinear study area, which can be roughly estimated by the aggregation of occurrences from a closely related taxon or a taxon group. However, such data or information are difficult to find for the native range of walnut or for that region as whole and a large spatial extent can also lead to the selection of a higher proportion of less informative background points [66]. Instead, we produced a bias grid by deriving a Gaussian kernel density map to be more selective in the choice of background points focusing on sampling locations of walnut. This method produces a bias grid that up-weights presence-only data points with fewer neighbors in the landscape; bias values of 1 reflect no bias while higher values indicate increased sampling bias [62, 64].

#### Tuning model settings

The unfiltered data with 237 data points and two filtered data with 136 and 112 occurrence points, were subjected to model tuning using an R package ENMeval [67] to identify the optimum data set for modeling current distribution of walnut. The ENMevaluate function in the ENMeval package performs tuning and evaluation of models by automatically implementing MaxEnt with a range of user-defined settings. It executes a series of tasks: (1) partitions occurrence and background data points into spatially independent evaluation bins using six different methods for *k*-fold cross validation [60, 68]; (2) builds a series of models with different user-specified feature classes (FCs) and regularization multipliers (RMs); and (3) computes five different evaluation metrics to aid in selecting optimum model settings. The evaluation metrics include: (i) the area under the curve (AUC) of the receiver operating characteristic (ROC) for the test data (AUC_TEST_ [60]); (ii) AUC_DIFF_ which is the difference between AUC_TRAIN_ and AUC_TEST_ [69]; (iii) minimum training presence omission rate (OR_MTP_ [60]); (iv) 10% training omission rate (OR_10_ [60]); and (v) the Akaike information criterion (AIC_c_ [69]). AUC_TEST_ measures the model’s ability to discriminate conditions at test occurrence localities from those at background localities averaged over *k* iterations, with higher values indicating better discrimination. AUC_DIFF_ is positively associated with the degree of overfitting. Omission rates provide information regarding the ability to discriminate between suitable and unsuitable sites as well as quantify model overfitting by comparing threshold-dependent omission rates with theoretically anticipated levels of omission. OR_MTP_ indicates the proportion of test localities with suitability values lower than those associated with the lowest-ranking training locality with values greater than zero typically indicating model overfitting. OR_10_ indicates the proportion of test localities with suitability values (relative occurrence rate corresponding to MaxEnt’s raw output) lower than those excluding the 10% of training localities with the lowest predicted suitability. Under either threshold rule, pixels with values equal to or higher than the threshold are considered suitable. Omission rates greater than the theoretical expectation for a given threshold typically indicate model overfitting. The AIC corrected for small sample size (AIC_c_) reflects both model goodness-of-fit and complexity, where the best model has the lowest value (i.e. ΔAIC_c_ = 0).

We applied the “block” method to partition both occurrence and background data, which splits data along the latitude and longitude lines, and allocates equally into four bins for cross validation. It is the best method for studies involving model transfer across space and time [70]. We built models with the RMs ranging from 1.0 to 5.0 at increments of 0.5 and six FC combinations: Linear (L); Linear and Quadratic (LQ); Hinge (H), Linear, Quadratic, and Hinge (LQH); Linear, Quadratic, Hinge, and Product (LQHP); and Linear, Quadratic, Hinge, Product, and Threshold (LQHPT) with 10000 background points. The RM imposes a penalty on model complexity and FC determines the shape of response curves, both act in concert with each other to reduce complexity of models. Computation of all evaluation metrics used MaxEnt raw output values, which is interpreted as relative occurrence rate (ROR) [71]. The model with ΔAIC_c_ equal to zero is considered the best model [69]. We computed Schoener’s D statistic that considers the geographic variability pixel-by-pixel to quantify pair-wise similarity among different models. Based on model tuning for different data sets, we selected the data set filtered at 10 km with 137 occurrence points as the best for hindcasting LGM and LIG distributions of walnut. We ran MaxEnt modeling with settings identified as optimum by model tuning to produce the current climatic projection and to hindcast past distributions of walnut with the Gaussian kernel density bias grid file to account for any residual sampling bias in the data set. Predicted habitat suitability maps for the current, LGM, and LIG distributions of walnut showing the relative rate of occurrence were generated in ArcMap 10.1.

## Results

### Genetic polymorphism and population structure

The walnut germplasm collection examined exhibited considerable polymorphism with observed number of alleles ranging from 8 for WGA089, WGA237, and WGA384 to 20 for WGA 202 with an overall mean of 12 alleles/locus (Table 1). The observed and expected levels of heterozygosity showed significant deficiency of heterozygotes for all loci as compared to Hardy-Weinberg expectations. The observed heterozygosity ranged from 0.326 for WGA349 to 0.651 for WGA178, with an overall mean of 0.501 and the fixation index, which indicates non-random assortment of alleles due to significant population sub-structuring, ranged from 0.136 for WGA178 to 0.610 for WGA349, with an overall mean of 0.285. Deficiency of heterozygotes is sometimes attributed to presence of null alleles, but their effects on population differentiation is not fully understood. The conventional methods for detecting null alleles are less reliable and inconsistent when applied to non-equilibrium populations, and provide only a sub-optimal solution [72].

**Table 1:** Locus-wise genetic variability in walnut germplasm.

Locus	*n*	*A*	*H*_*e*_	*H*_*o*_	*F*
WGA001	702	13	0.810	0.605	0.253
WGA004	666	12	0.716	0.536	0.252
WGA009	662	12	0.762	0.650	0.148
WGA069	668	14	0.830	0.603	0.273
WGA089	709	8	0.682	0.495	0.274
WGA106	702	7	0.422	0.339	0.196
WGA118	701	16	0.743	0.615	0.173
WGA178	653	13	0.753	0.651	0.136
WGA202	675	20	0.841	0.631	0.250
WGA223	674	15	0.798	0.527	0.341
WGA225	657	10	0.475	0.327	0.311
WGA237	687	8	0.601	0.412	0.315
WGA318	651	19	0.864	0.399	0.538
WGA321	701	12	0.712	0.589	0.173
WGA331	687	9	0.631	0.408	0.354
WGA332	694	11	0.660	0.532	0.194
WGA338	693	9	0.509	0.427	0.161
WGA349	644	19	0.836	0.326	0.610
WGA384	676	8	0.659	0.448	0.320
Mean	679	12	0.700	0.501	0.285

*n* = Average samples size, *A* = Alleles/locus, *H*_*e*_ = Expected heterozygosity, *H*_*o*_ = Observed heterozygosity, *F* = Fixation index

**Fig 1:**
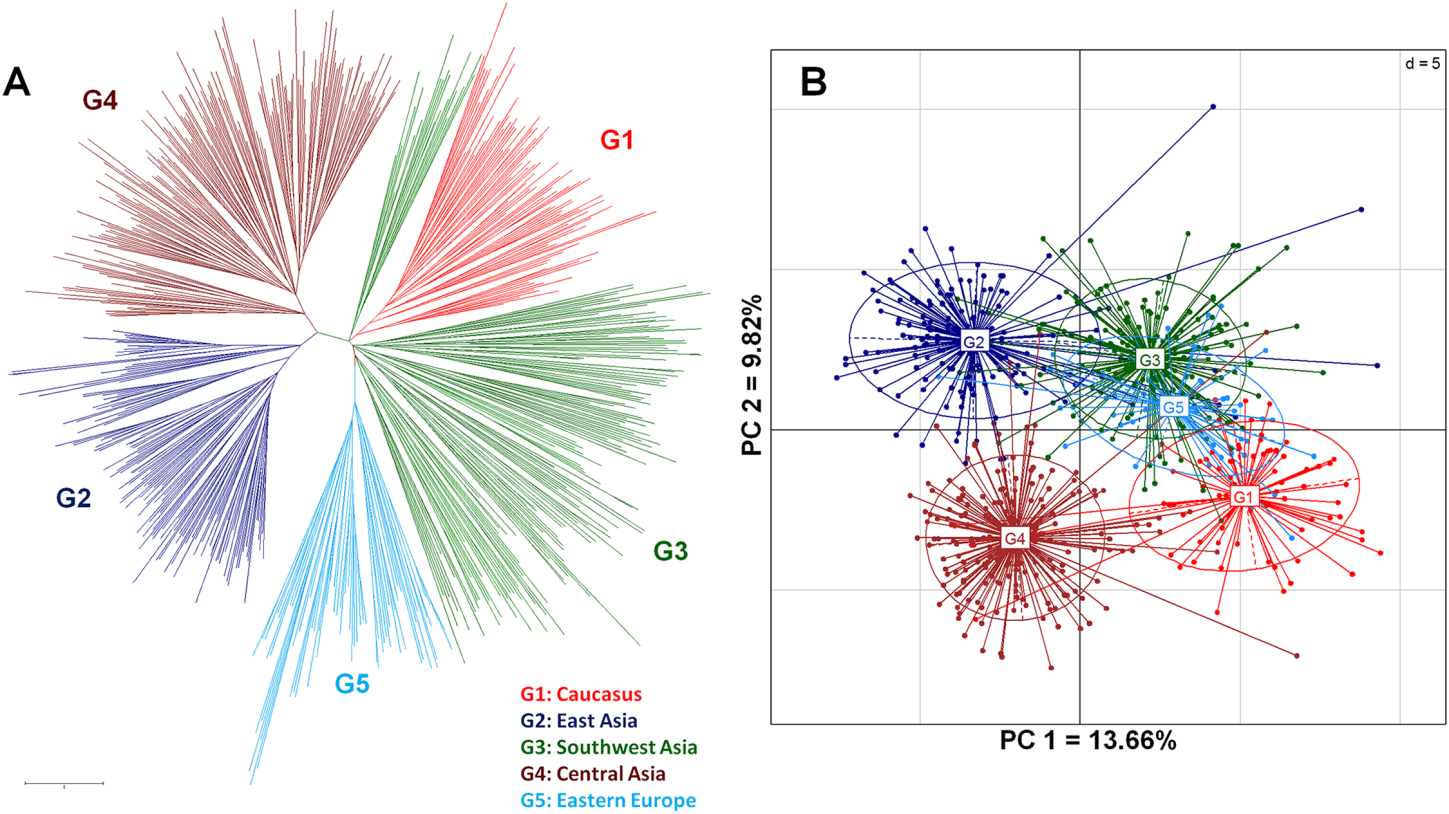
Genetic relationships among walnut genotypes. (A) Neighbor-joining cluster analysis using pair-wise Nei and Li distance matrix. (B) Principal components analysis using multilocus microsatellite genotype data.

Multivariate genetic structure revealed by the CA identified five major groups closely matching with the geographic affiliations of different walnut accessions (Fig 1A). Eastern European accessions from the Balkans, Carpathians, Russia, western Europe mainly French showed close genetic affinity with the SW Asian and the Caucasus groups. East Asian accessions from China and the Central Asian germplasm from Kyrgyzstan formed two unique groups somewhat allied to each other. The SW Asian germplasm from Afghanistan and neighboring Tajikistan, India, Nepal, and Pakistan formed a loose conglomeration exhibiting subtle differentiation among them. The Transcaucasian germplasm from Azerbaijan and Georgia formed an exclusive group closely associated with the SW Asian group.

The PCA based on mutlilocus genotype data unraveled genetic relationships within and among different geographic groups similar to CA. The two-dimensional projection of accessions defined by the first two principal axes accounting for 13.66% and 9.82% of the total variation, respectively, revealed genetic differentiation within and among groups (Fig 1B). The first axis discriminated the Central Asian and East Asian groups from the SW Asian, Caucasian, and Eastern European groups, whereas the second axis differentiated the East Asian from the Central Asian group and among the Caucasus, Eastern European, and the SW Asian groups.

**Fig 2:**
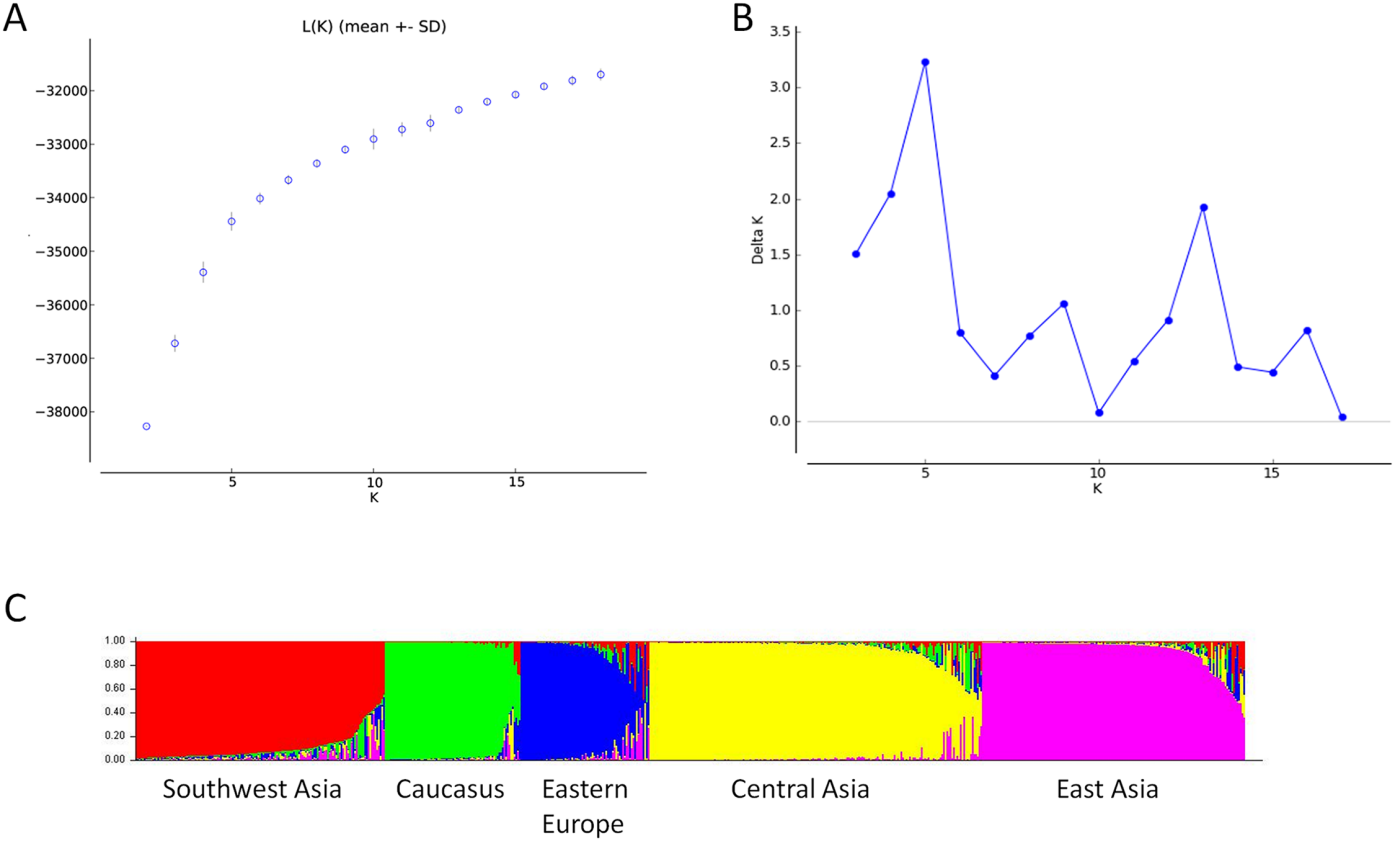
Population structure inferred from a model based Bayesian cluster analysis. (A) Posterior probabilities (Ln Pr *X*|*K*) averaged over 20 replicate runs, (B) The *ad hoc* statistic delta *K* related to the second order rate of change of log probability of data between successive values of *K* with a distinct peak at *K* = 5 with some minor peaks at *K* = 9, 13, and 16, and (C) Bayesian Inferred population structure of walnut for *K* = 5 groups.

The model-based Bayesian CA produced results comparable to the distance based CA and PCA. The estimated mean likelihood values (Ln Pr *X*|*K*) attained a maximum value at *K* = 5 (Fig 2A). The *ad hoc* statistic Δ*K* related to the second order rate of change of log probability of data between successive *K*s produced a distinct peak at *K* = 5 with some minor peaks at *K* = 9, 13 and 16 (Fig 2B). Plotting the *Q*-matrix of estimated membership coefficients for each individual genotypes for *K* = 5, sorted by *Q* revealed clusters somewhat similar in size and composition to distance based CA and PCA (Fig 2C). However, genotypes with mixed ancestry, often involved members from each of the five geographic groups of walnut.

### Pattern of distribution of genetic diversity within and among geographic groups

The contingency *χ*^2^ analysis indicated that the five geographic groups differed significantly in the number, composition, and frequency of alleles. However, there were a number of high frequency alleles common across the groups that often possessed frequencies lower than 0.1 in some groups. There were 87 unique low frequency alleles among groups with the SW Asian group possessing the largest number with 50 unique alleles followed by East Asia with 20, Central Asia with nine, the Caucasus with six and the Eastern European group with two (S4 Table).

**Fig 3:**
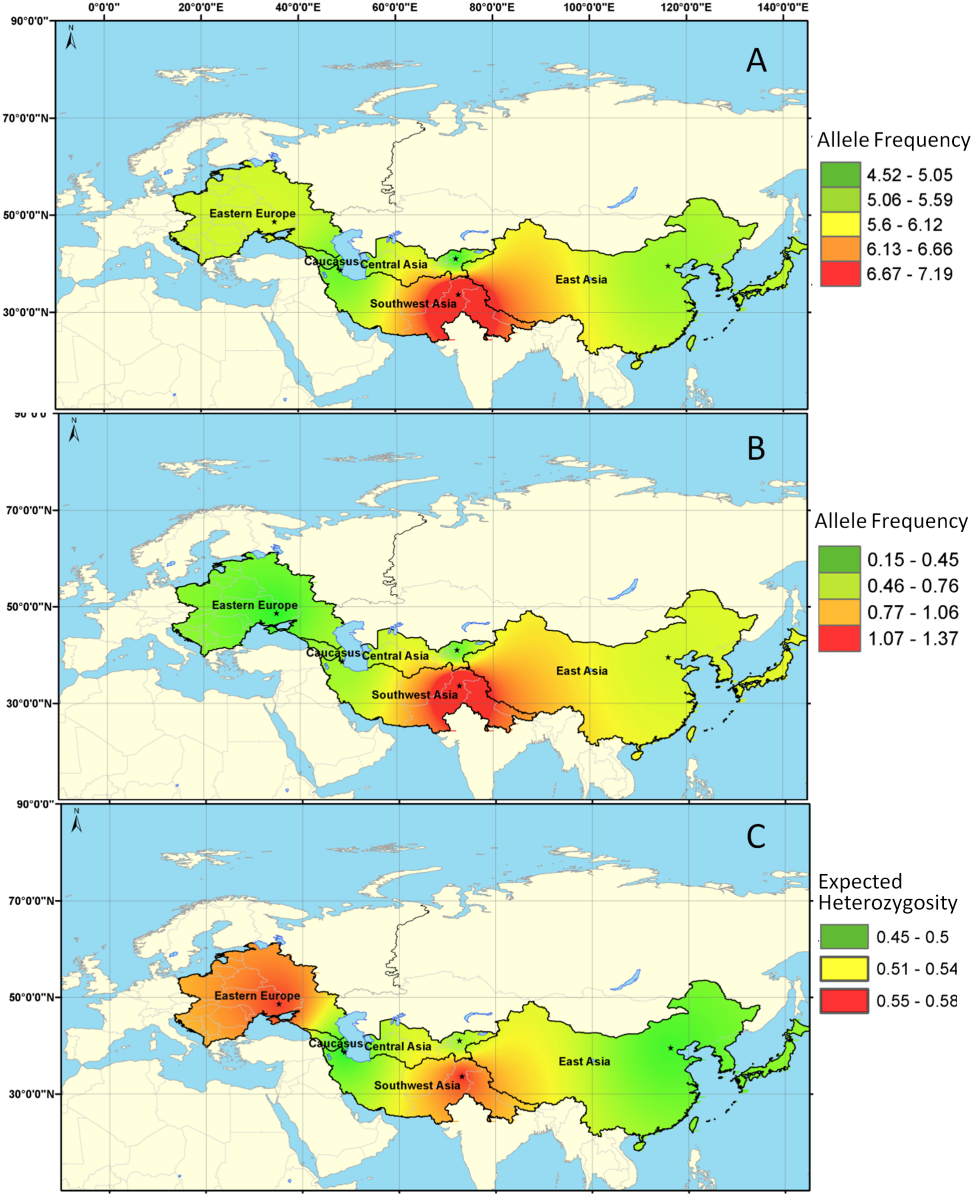
Inverse distance weighted (IDW) interpolation of allelic diversity estimates in walnut. (A) allelic richness, (B) private allelic richness, and (C) expected levels of heterozygosity among walnut geographic groups.

Estimates of within-group diversity parameters indicated that the total number of alleles across 19 loci ranged from 191 with a mean of 10.1 alleles/locus for the SW Asian group to 100 with a mean of 5.26 alleles/locus for the Caucasus. The allelic richness adjusted to the minimum sample size of 49 genes ranged from 7.19 for the SW Asian group to 4.52 for the Central Asian group with an average of 5.29 alleles/locus and the private allelic richness followed the same trend (Table 2, Fig 3A and 3B). There was a deficiency of heterozygotes in all the five groups suggesting moderate levels of population subdivisions within groups. Partitioning of variation within and among geographic groups indicated that most of the molecular variation (87%) resided within populations and only 13% of the total variation accounted for genetic differentiation among groups (Table 3). The estimated degree of among-group differentiation (*F*_*ST*_) averaged over loci among groups was 0.128 (*P* < 0.01).

**Table 2:** Within-group genetic variability in walnut.

Population	*n*	*A*	*Ar*	*PAr*	*H*_*e*_	*H*_*o*_	*F*	Total Alleles
Caucasus	68.7	5.263	4.660	0.340	0.602	0.453	0.249	100
East Asia	142.2	6.790	4.870	0.480	0.564	0.451	0.201	133
Southwest Asia	154.2	10.053	7.190	1.370	0.727	0.584	0.198	191
Central Asia	183.3	6.316	4.520	0.210	0.596	0.483	0.190	120
Eastern Europe	48.6	5.684	5.190	0.150	0.678	0.582	0.143	108
Mean	113.2	6.684	5.286	0.510	0.623	0.502	0.195	130

*n* = Average samples size, *A* = Alleles/locus, *Ar* = Allelic richness, *PAr* = Private allelic richness, *H*_*e*_ = Expected heterozygosity, *H*_*o*_ = Observed heterozygosity, *F* = Fixation index

**Table 3:** Partitioning genetic variation within and among geographic groups in walnut.

Source of variation	d.f.	Sum of squares	Variance components	Percentage variation
Among Groups	5	424.430	0.36063**	12.83
Within Groups	1426	3495.415	2.45120	87.17
Total	1431	3919.844	2.81184	

Fixation Index *F*_*ST*_: 0.12826

**Significant at P<0.01 based on 1023 permutations

Nei’s gene diversity analysis based on allele frequencies for the five groups identified from the CA indicated that the total gene diversity, a measure of heterozygosity in the total population is reasonably high across loci ranging from 0.418 for WGA106 to 0.873 for WGA349 with an average of 0.706. Only 12.4% of the total gene diversity (*G*_*GT*_) accounted for genetic differentiation among groups and there was considerable variation among loci ranging from 8.3% for WGA331 to 22.2% for WGA384, and on average 88% of the total variation was found within group variation (Table 4).

**Table 4:** Genetic differentiation among geographic groups in walnut.

Locus	*H*_*T*_	*H*_*G*_	*D*_*GT*_	*G*_*GT*_	Within Group
WGA001	0.811	0.734	0.077	0.095	0.905
WGA004	0.688	0.617	0.071	0.103	0.897
WGA009	0.770	0.687	0.082	0.107	0.893
WGA069	0.829	0.767	0.062	0.075	0.925
WGA089	0.684	0.572	0.112	0.163	0.837
WGA106	0.418	0.381	0.038	0.090	0.910
WGA118	0.781	0.675	0.106	0.136	0.864
WGA178	0.749	0.694	0.055	0.073	0.927
WGA202	0.857	0.766	0.091	0.106	0.894
WGA223	0.809	0.719	0.090	0.111	0.889
WGA225	0.532	0.471	0.061	0.115	0.885
WGA237	0.587	0.511	0.076	0.130	0.870
WGA318	0.857	0.671	0.185	0.216	0.784
WGA321	0.746	0.671	0.074	0.100	0.900
WGA331	0.650	0.596	0.054	0.083	0.917
WGA332	0.659	0.581	0.078	0.119	0.881
WGA338	0.496	0.438	0.058	0.118	0.882
WGA349	0.873	0.726	0.147	0.169	0.831
WGA384	0.625	0.486	0.139	0.222	0.778
Mean	0.706	0.619	0.087	0.124	0.876

*H*_*T*_ = Total gene diversity, *H*_*G*_ = Gene diversity within groups, *D*_*GT*_ = Gene diversity between groups, *G*_*GT*_ = Proportion of gene diversity due to differentiation

Geographic differentiation among the walnut groups was estimated using Wright’s fixation index (*F*_*ST*_) (Table 5). The Caucasus group exhibited the highest divergence from the East Asian group (0.165) followed by the Central Asian group (0.158), the Eastern European group (0.116), and the SW Asian group (0.09). The SW Asian group is closely related to the rest of the groups in the study with *F*_*ST*_ ranging from 0.045 with the Eastern European group, followed by the Caucasus and the East Asian groups (0.09 each) and the Central Asian group (0.103).

**Table 5:** Pair-wise *F*_*ST*_ values showing genetic differentiation among geographic groups in walnut.

	1	2	3	4	5
Caucasus	0.000				
East Asia	0.165	0.000			
SW Asia	0.090	0.091	0.000		
Central Asia	0.159	0.119	0.103	0.000	
Eastern Europe	0.116	0.122	0.046	0.099	0.000

### Ecological niche modeling

**Fig 4:**
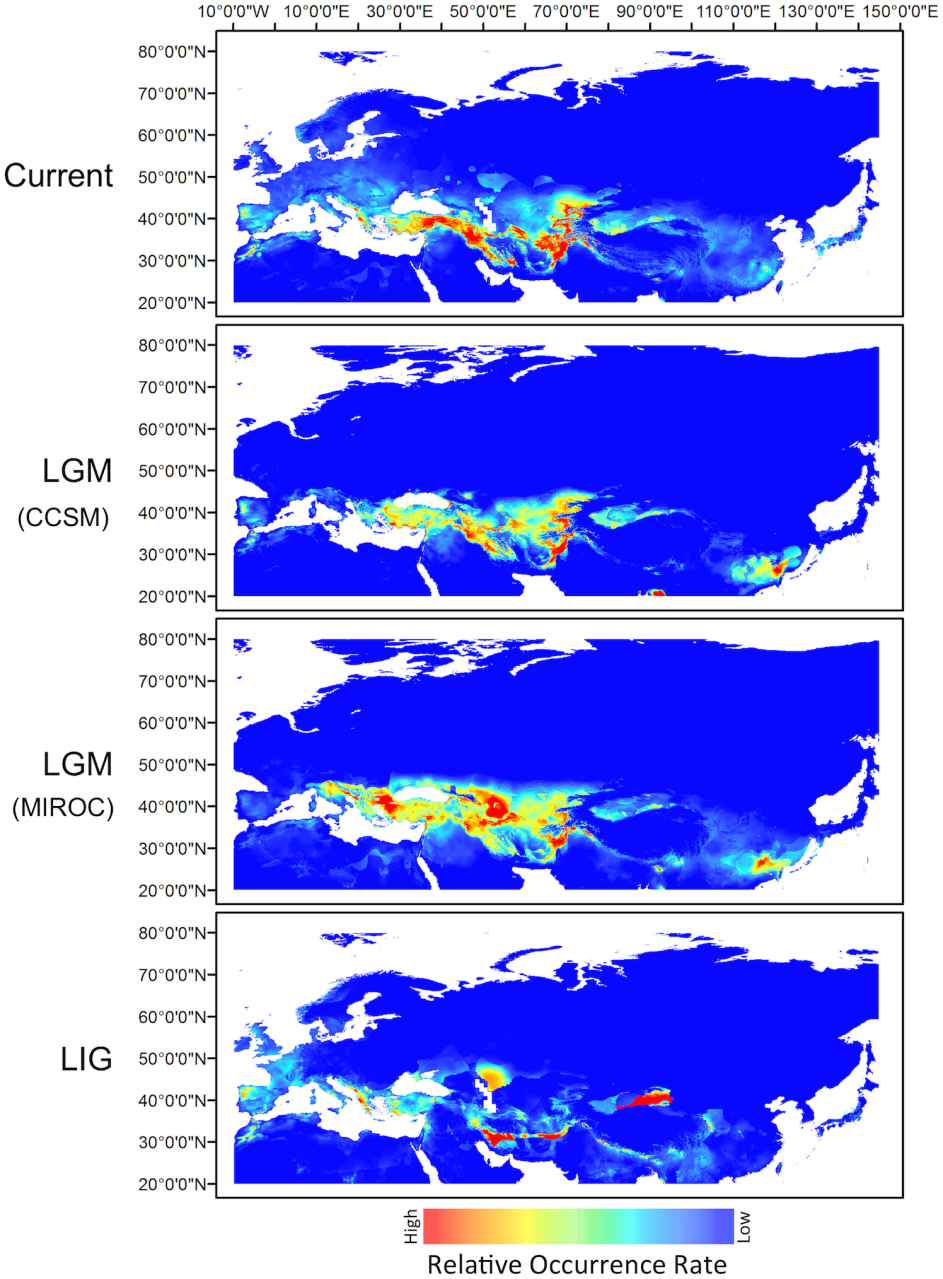
Ecological niche modeling of walnut distributions. AIC_c_-selected model prediction of occurrence of walnut for current, last glacial maximum (LGM; 21–18 kyr BP), and last interglacial (LIG; 130–107 kyr BP) climatic conditions for the data set filtered at 10 km with 137 occurrence points (refer to Table 6 for feature class and regularization multiplier settings).

Model tuning results are presented in S5 Table, S1 Fig, and summarized in Table 6. Examining the metrics of AIC_c_-selected models suggested that the data set filtered at 10 km logged in the lowest values for AUC_DIFF_ (0.014), OR_MTP_ (0.029) and OR_10_ (0.103) among the three models followed by the unfiltered data set with 0.029, 0.042, and 0.118, and filtered at 25 km with 0.040, 0.065 and 0.185, respectively, suggesting filtering somewhat improved model efficiency. Visual examination of models generated from hindcasting LGM and LIG distribution of walnut using the three data sets (S2 Fig, Fig 4) showed minor difference among the projections suggesting filtering did affect only marginally the LGM and LIG predictions and Schoener’s D statistics further confirmed these results (Table 7). Based on evaluation metrics, we selected the model from the data set filtered at 10 km (Fig 4) to hindcast LGM and LIG walnut distribution.

**Table 6:** Model settings (evaluation metrics) for AIC_*c*_-selected MaxEnt model predictions.

Data set	Data points	FC	RM	AUC_TEST_	AUC_DIFF_	OR_MTP_	OR_10_	ΔAIC_c_
Unfiltered	237	LQHP	4	0.9402	0.029	0.042	0.118	0
Filtered at 10 km	137	LQHP	5	0.9358	0.014	0.029	0.103	0
Filtered at 25 km	112	LQHPT	3.5	0.9095	0.041	0.065	0.185	0

FC = Feature class; RM = Regularization multiplier; AUC_TEST_ = Area under the curve (AUC) of the receiver operating characteristic (ROC) for the test data; AUC_DIFF_ = AUC_*TRAIN*_ − AUC_TEST_; OR_MTP_ = Minimum training presence omission rate; OR_10_ = 10% training omission rate; ΔAIC_c_ = Akaike information criterion

**Table 7:** Pair-wise Schoener’s D statistic measuring niche similarity among the current, LGM, and LIG model predictions.

	Data set		
Model/Data set	Unfiltered	Filtered at 25 km	Filtered at 10 km
CURRENT			
Unfiltered (237 data points)	1	0.8069	0.9381
Filtered at 25 km (137 data points)		1	0.8173
Filtered at 10 km (112 data points)			1
LGM-CCSM			
Unfiltered (237 data points)	1	0.8117	0.9352
Filtered at 25 km (137 data points)		1	0.8298
Filtered at 10 km (112 data points)			1
LGM-MIROC			
Unfiltered (237 data points)	1	0.8052	0.9322
Filtered at 25 km (137 data points)		1	0.8141
Filtered at 10 km (112 data points)			1
LIG			
Unfiltered (237 data points)	1	0.7267	0.8842
Filtered at 25 km (137 data points)		1	0.7459
Filtered at 10 km (112 data points)			1

The current climatic model predicts a moderate to high rate of occurrence of walnut in the regions mainly between 30°N to 45°N latitude, and 20°E to 80°E longitude, comprising eastern Turkey bordering the Black Sea and western Iran, the Talysh region of Azerbaijan, southern Turkmenistan, western Uzbekistan, Kyrgyzstan, southern Kazakhstan, Tajikistan, northern Afghanistan, northwestern Pakistan extending into southeastern regions, southcentral Tibet, and northeastern India. Parts of western and central Turkey, the Balkan Peninsula extending into eastern Greece and southern Bulgaria, southeastern Carpathians (Romania), and northeastern Danube region (Hungary, Slovakia, Czech Republic, Austria), Spain, the Atlas Mountains of North Africa, western China (Xinjiang Province), parts of northern China bordering Mongolia (Shanxi, Hebei, Henan and Shaanxi areas), and southeastern China in the Fujian and Guangxi provinces, showed relatively low rate of occurrence of walnut. Some scattered areas of northern and southern Turkey, southeastern Adriatic Sea coastal region including northwestern Greece, western Albania and northwestern Spain bordering Portugal, showed relative high rate of occurrence of walnut. Overall, the current climatic model roughly predicted the current natural distributional range of walnut (Fig 4).

The LGM-CCSM projection predicted the areas of relatively high rate of occurrence of walnut shifted to lower latitudes than projected in the current climatic model. Distribution was fragmented and interspersed with areas of marginal occurrence. Eastern Pakistan extending in the north to Tajikistan and parts of northeastern Afghanistan, southeastern Turkmenistan, western Iran, southern Turkey bordering the Mediterranean Sea, the Hyrcanian and Colchic regions of the southern Caucasus including the Talysh and Alburz mountain ranges of Azerbaijan and Iran, Armenia and border areas of the Black Sea, showed relatively high rates of occurrence of walnut. However the entire Turkey, southern Balkans and eastern coastal regions of Adriatic Sea and in Central Asia, southern Turkmenistan, Uzbekistan down to Tajikistan, northeastern Afghanistan, and western Himalayan state of Kashmir extending up to southwestern Tibet exhibited moderate to low rates of occurrence. The LGM-MIROC model projected a similar distribution as LGM-CCSM, but regions of high relative occurrence concentrated in north eastern Pakistan, Tajikistan, northeastern Afghanistan, southern Turkmenistan on eastern coast of Caspian Sea, southwestern Balkans (southern Bulgaria), northwestern Turkey, north western coastal regions of the Adriatic Sea. Both CCSM and MIROC models projected a moderate rate of occurrence of walnut in Xinjiang province of western China, low occurrence in central China and relatively high occurrence rate in the southeastern China (Fujian and Jiangxi provinces and neighboring areas), and somewhat fragmented distributions in northeastern India. There were regions of low occurrence in central China, but overall there was a reduction in the occurrence of walnut in East Asia during LGM, as compared to the present day distribution. The LIG projection indicated relatively high rates of occurrence in a narrow region comprising southern Iran and northwestern Pakistan extending into southern Afghanistan, tapering off eastward along the southern Himalayan foothills extending into Nepal, Sikkim, Bhutan into Arunachal and north Assam. There was also a region of high occurrence in the northeastern and central regions of Xinjiang province. There was a narrow region of relatively high occurrence in Shaanxi and Shanxi provinces. Surprisingly the southeastern region of high occurrence in China seen during LGM was not obvious during the LIG. Eastern Kazakhstan bordering the northern Caspian Sea showed a moderate rate of occurrence, while southeastern Turkey along Mediterranean coast, eastern coastal regions of Greece, Albania, and Montenegro showed high rates of occurrence. There was an incidence of extended low occurrence throughout Western Europe including Spain, Portugal, France, and Belgium extending into Germany and Parts of the United Kingdom, except for a small northeastern region of Spain above Portugal showed high occurrence. Morocco along the Atlas Mountains showed moderate occurrence of walnut during the LIG.

## Discussion

The distribution and survival of trees during the LGM has been of interest to paleoecologists, biogeographers, and geneticists. Paleodistribution modeling in conjunction with population genetic analyses can predict the past distributions and aid in locating Pleistocene refugia of plant species. Ecological niche models (ENMs) that associate species occurrence and abundance with climatic variables are extensively used to gain ecological and evolutionary insights, and to predict species distributions across landscapes over space and time. The present study deals with the glacial history of walnut to address questions related to past distributions during the LGM and LIG periods. The results include population genetic analysis of a germplasm collection representing the modern range of walnut, and ecological modeling of present distribution and the LGM and LIG projections, to predict past climatic niches and locate the Pleistocene refugia.

### Historical biogeography and glacial history of walnut

Walnut is considered a Tertiary relict, native to a broad geographic area extending from the Near East through Central Asia to the Himalayas and Western China [36, 73]. Zeven and Zhukovsky [37] consider Central Asia and adjacent Near Eastern regions as the primary centers of origin and diversity of walnut. Several lines of fossil evidence support that ancestral forms of walnut were widespread throughout Eurasia during the Miocene [20, 22, 74–78]. The earliest evidence of macrofossils of *J. accuminata*, similar to the present day walnut, comes from Europe and the Caucasus dating back to the Miocene and Pliocene [27, 74, 77]. Pollen and macro-fossils of walnut were also reported from several European locations in central France, England, Belgium, The Netherlands, northern Italy, and Spain, extending eastward into southern Asia including Tibet, from the late Tertiary through the Quaternary [22, 73] approximately matching the estimated time of divergence of the section *Juglans* within the genus *Juglans* [17].

It is widely accepted that during the LGM, most nemoral tree species were restricted to refugia in the Iberian, Italian, and Balkan peninsulas [79–81]. However, during the interglacial stages of the lower Pleistocene southeastern Europe supported extensive mixed-broad leaved forests of *Fagus*, *Juglans*, *Pterocarya*, and *Tsuga* south of 57°-58°N [82, 83], as the climate deteriorated the proportion of broad-leaved species was reduced and eventually eliminated. During the Eemian interglacial (130 -116 Kyr BP), it is generally believed that the thermal optimum was higher than today, and the dendroflora pollen spectra in the vicinity south of the Gulf of Finland and Central Europe supported broad leaved deciduous species such as *J. regia*, *Carpinus betulus*, *Tilia cordata*, *T. tomentosa*, *Quercus* spp. *Corylus avellana* and *Alunus* spp. [84, 85]. Fossils of walnut were also discovered in Bilzingsleben, a Paleolithic site in Germany dating back to the Eemian interglacial period, indicating walnut persisted until the Ionian stage of the middle Pleistocene [85]. There is palynological evidence of existence of walnut in the Balkan refugia during the LGM but it is interpreted either as representing long distance dispersal from southern refugia, or as *in situ* refugia [86]. The LGM-MIROC model (Fig 4) in our study strongly supports a high rate of occurrence of walnut in the southern Balkan regions of Bulgaria and Romania adjacent to the Black Sea coast. However, the Holocene landscape comprising *Juglans*, *Castanea*, *Platanus*, *Olea* and *Fagus* is thought to be anthropogenic intervention [32, 87–89] during the Greco-Roman period.

During pre-glacial periods the section *Juglans* endemic to Eurasia probably had ample opportunity to diversify and much of the ancestral taxa must have gone extinct during glaciations. Incomplete palaeobotanical records from Eurasia and perhaps the difficulty in recognizing intrasectional diversity in pollen and other microfossil flora have obscured the ancestral taxonomic diversity of the section *Juglans*. However, palynological evidence suggests that walnut survived in Central Europe in small cryptic refugia during the LIG [11, 84–86] and gradually became extinct [90] during the LGM. In contrast, the southern Caucasus and SW Asia have sheltered a large number of Tertiary relict nemoral trees, including walnut, during the LGM [77, 91–93]. Our ENMs suggest that walnut probably had multiple refugia spread out from the southern Caucasus to Southwestern and Central Asian regions surrounding the Pamir Mountain ranges, where more favorable Pleistocene and early Holocene climates prevailed in most of Eurasia (Fig 4).

### Glacial refugia, postglacial recolonization, and genetic differentiation

The climatic deterioration during the late Tertiary followed by the Quaternary glacial and intergalcial fluctuations played a major role in shaping the present-day genetic diversity, population structure, and differentiation patterns of plant species [2, 15, 94]. Whether Quaternary vegetation dynamics fostered increased or decreased genetic diversity is unknown, but demographic fluctuations during range expansion and contraction could cause undesirable stochastic effects resulting in widespread extinctions [95]. However, the genetic signatures of historical biogeographic events persist long after post glacial recolonization from refugial populations [95, 96]. Glacial refugia were important for species survival in glacial and interglacial periods and sheltered many species which had been widespread [15]. Knowledge of the size, distribution, isolation within and among refugia, and the mode of postglacial expansion are important to understanding the mode and tempo of evolution of modern day species [90]. Therefore, postglacial expansion of species is an important issue in the study of historical biogeography of the Quaternary [97]. It has been shown that postglacially colonized regions are known to exhibit lower genetic diversity than refugia [2, 97], and as expansion proceeds its leading edge will have progenies from the nearest neighborhoods as compared to the ones far behind [98]. As colonization continued, the natural selection, local adaptation and gene flow within and among the new neighborhoods and populations from different refugia will eventually build dynamic species-wide population genetic structure. Consequently the study of contemporary genetic structure of species populations should be able to shed light on past glacial events that shaped genetic diversity and modern distribution of species aiding in identification of areas where species may have survived glaciations. Here we test whether or not the amount and pattern of distribution of genetic diversity within and among different geographic groups of walnut shed light on the Pleistocene glacial history and the postglacial re-colonization, domestication and distribution.

Humans apparently played a role in shaping the modern genetic structure, but the signature of biogeographic events should permit speculation on the mode and tempo of the evolution of walnut [99–101]. Our study of genetic diversity suggests five genetic groups reflecting regional centers of genetic diversity and differentiation, and we hypothesize that these groups embody the biogeographic history of walnut. Among the groups, SW Asian walnuts from the regions of Afghanistan, Pakistan, southern Tajikistan and parts of northwestern India represented the most diversity as indicated by high levels of allelic richness, private allelic richness, and heterozygosity, followed by groups from Eastern Europe, East Asia, and the Caucasus (Table 2 and Fig 3). This suggests that walnut may have survived in SW Asia during the LGM and served as a founder for recolonization of neighboring Central Asia, the Caucasus, East Asia, and Eastern Europe during the current Holocene interglacial. The LGM-CCSM and LGM-MIROC projections (Fig 4) show high occurrence of walnut interspersed with regions of moderate to low occurrence indicating a mosaic of isolated populations thriving in South and Southwest Asia during the LGM. Hemery et al. [102] suggested that walnuts may have migrated northward towards Central Asia from South Asia sometime during the Holocene. Beer et al. [99] proposed expansion of walnut from South and SW Asia to Central Asia during Chalcolithic period based on palynological data.

A significant amount of genetic diversity was detected in the walnut germplasm collection, and the loci assayed differed considerably for the number of alleles per locus, observed and expected levels of heterozygosity, and fixation index (Table 1). General deficiency of heterozygotes and relatively high fixation index across loci is attributed to the Wahlund effect caused by significant intra- and inter-regional genetic differentiation, which is perhaps due to sampling effect in germplasm collections of outbreeding species like walnut. Finite and isolated populations in the mountainous terrains where walnut is native probably experienced drift leading to stochastic loss and/or fixation of alleles.

Central Asian walnuts show the lowest level of allelic richness and low heterozygosity as expected in recently colonized populations. Moderate allelic richness and heterozygosity of Eastern European walnut observed here is unexpected and probably due to historic migration of germplasm, recent introductions from other walnut growing regions, and directional selection during domestication that occurred in this region compared to other walnut regions. Surprisingly, the East Asian walnut exhibited moderate allelic richness and private allelic richness compared to the Transcaucasian and Central Asian walnuts, probably due to historic introductions of diverse germplasm from Persia, Tibet, and India [35], and possible interspecific gene flow between walnut and its native butternut counterparts, which were prevalent in northeastern China. The low allelic richness within the Caucasus walnuts may be due to severe bottleneck within and among the fragmented populations growing in diverse topographic, pedological, temperature and moisture conditions eroding the allelic diversity [103]. Human habitation and expansion of agriculture in this region during the late Pleistocene and Holocene have caused profound changes in soil cover and vegetation on a vast geographic scale impacting ecosystems in the southern Caucasus. Further, over harvesting and grazing in walnut forests, and more recently the forest farming systems have hampered regeneration and fragmented walnut distribution, eroding the genetic diversity and promoting differentiation among populations in the Caucasus. A recent study showed significant genetic differentiation among moderately variable fragmented walnut populations in the greater and lesser Caucasus Mountain ranges [104].

The CA and PCA results suggest close association of the SW Asian, Caucasus, and Eastern European walnut groups, while Central Asian and East Asian walnut are somewhat separate groups. Presence of one or more moderate to high frequency alleles common across loci and among groups and low differentiation of SW Asia walnut from other groups indicate that walnut probably expanded from SW Asia into other regions following glaciations. At the same time, the presence of several moderate frequency alleles across loci restricted to one or more groups suggest either local genetic differentiation after recolonization or separate expansion events from different refugia. However, high genetic diversity and close genetic affinity of SW Asian walnut strongly support a single refugial source located in the mountainous regions of SW Asia during the LGM that further expanded and spread northward into Central Asia and westward into Europe and other regions, which was further facilitated by human migration along ancient trade routes. Furthermore, human mediated dispersal and local domestication events since Greek and Roman times perhaps significantly influenced the current distribution of genetic diversity in walnut. Historic migration of walnut along the ancient trade routes from Persia, Tibet, and the Himalayan regions of India into China during the Han dynasty founded an important secondary center of diversity for walnut [35].

The two likely scenarios for recolonization of trees: (1) rapid colonization from southern refugia mediated by long-distance dispersal [105], which is unlikely as walnut is mainly dispersed by small mammals and birds and (2) slow dispersal from wide-spread refugia with some closely located to the modern range [106]. The latter is more likely as our results indicate that post-glacial spread of walnut probably occurred gradually to neighboring Central Asia, the Caucasus, East Asia and then to Eastern Europe. Our LGM and LIG models indicate the possibility of the Balkans, Caucasus, Central Asia and neighboring regions also supporting glacial refugia which may have contributed to rapid postglacial colonization of walnut. Further, it is widely believed that the post-glacial colonization of nemoral Europe comes from one or both southern refugia; Caucasus, SW Asia. Despite our genetic analysis supporting SW Asian walnut as a single founder source for post-glacial recolonization, the ENMs suggest the possibility of many more refugia in the Balkans, southern Caucasus, west, central, and south Asian regions. Our LIG projection (Fig 4) supports widespread but fragmented and low rate of occurrences of walnut throughout southern and western Europe as far north as southern Scandinavia, southern Ukraine, the coastal Adriatic regions of Greece and Albania, extending east into Turkey, southwestern Kazakhstan, eastern Iran, northeastern Pakistan, southern Tibet, and foothills of the Himalayas extending into northeastern India (Sikkim and Arunachal), and Bhutan. There were isolated populations of low to medium occurrences in northern Afghanistan and northern Pakistan and it was missing in Central Asia and southern Caucasus. Expansion and contraction of walnut populations during the Pleistocene interglacials probably resulted in isolation of subpopulations within and among regional groups as evidenced by the significant deficiency of heterozygotes and inbreeding coefficients for all groups across loci contributing to moderate differentiation within groups. The Bayesian CA exhibited subtle differentiation among the five groups showing genetic admixture, which is probably due to shared ancestral polymorphisms or recent dispersal mediated by human migration along the silk routes and gene flow between bordering populations. Eastern European walnuts showed a greater percentage of admixture suggesting the strong influence of historic introductions and human selection.

### Possibility of multiple southern refugia

The presence of the Plio-Pleistocene cryptic refugia in regions other than SW Asia is not ruled out as they present moderate levels of genetic variation and differentiation within each group. In the Caucasus, the Colchis and Talysh regions served as species-rich refugia for many members of the Arcto-Tertiary flora where perhaps walnut survived during the glaciations [77, 103]. The Caucasus had much more favorable Pleistocene and early Holocene climates than most of Eurasia with its complex topography providing diverse habitats and isolation favoring the formation of refugia in which ancient species survived Pleistocene climate deterioration [77, 92]. The first fossil remains of walnut in Georgia date back to the Paleocene and the Sarmatian flora, a Miocene relict flora of Abkhazia (Colchis) somewhat similar to present flora containing subtropical elements such as walnut [107], where it remained dominant until the Early Pleistocene. Walnut currently survives in small isolated populations and in planted stands throughout Transcaucasia. In a recent study we showed limited diversity and significant differentiation among the walnut populations from the Talysh Mountains in the Lesser and Greater Caucasus Mountains [104].

Central Asian Mountain ranges such as the Pamir, Kopet Dagh, and Tien Shan are important centers of biological diversity and believed to be a center of origin and diversity of walnut [108–110]. The Kopet Dagh riparian forests along the southern and southwestern shores of the Caspian Sea, which to some degree resemble the Hyrcanian forests, where walnut has been reduced to sparse isolated populations from over harvesting and intense grazing [111]. The northwest Pamir Mountains of southern Tajikistan, especially the Gissar, Darvaz and Peter the First Ridges, support mesophyllic forest ecosystems consisting of walnuts (*J. regia* and *J. fallax*) and willow-poplar-birch forests at altitudes of 1000 to 1400 m and are considered to be relict formations of the Iranian and Turanian floras with eastern Mediterranean species occurring within distinct areas [112]. The walnut forest of western Kyrgyzstan is considered a Tertiary relict [110], but a recent palynological study indicated that it is probably of anthropogenic origin and at most 2000 years old [99]. Our analysis indicates that the Central Asian walnuts from the Fergana and Chatkal Ranges of Kyrgyzstan intergrade into the East Asian group forming a loose alliance with SW Asia and the Caucasus walnut groups (Fig 1). These results combined with our ENMs appear to suggest that walnut possibly (1) survived in small populations during glaciations in the Tien Shan Mountains extending up to the Fergana Ridge and southern Kazakhstan, (2) spread from the South and SW Asia into Central Asia following glaciation, and (3) survived in multiple refugia in many southern locations during glaciations. The LGM projections also suggest a low rate of occurrence indicating possible refugia of walnut in northeastern Xinjiang province and southeastern China. However, the high genetic variability and close genetic affinity of SW Asian walnuts to the Caucasus, East Asian, Eastern European, and Central Asian groups strongly suggest that walnut survived in SW Asia during glaciations (Tables 2 and 5). SW Asia served as a founder to post glacial range expansion of walnut into neighboring Central Asia, the Caucasus, and East Asia probably occurred during the Holocene. Strikingly, Eastern European walnuts showed closer relationship to the SW Asian and Central Asian groups, suggesting historic and repeated introductions of walnut from Asia into Southern and Eastern Europe during the Greco-Roman period and human selection and cultivation of these early Asian introductions. Further, ancient Chinese records indicate walnut was introduced to China from Iran, Tibet, and Kashmir region of India during the Han Dynasty [35]. This is further substantiated by *F*_*ST*_, which suggests that the East Asian group exhibits closer affinity to the SW Asian group than the other four regional groups, perhaps suggesting historical migration of walnut from this region through early trade along the ancient silk route connecting these two regions. Nonetheless, during the last glacial maximum, at least two independent refugia were maintained across northeastern China for *J. mandshurica*, a species representing the section *Cardiocaryon* within the genus *Juglans* [113].

### Ecological niche models

Ecological niche modeling of current climatic and species occurrence data predicted southern Caucasus, parts of West and Central Asia extending into South Asia encompassing northern Afghanistan, Pakistan, northwestern Himalayan region, and southwestern Tibet as the favorable climatic niche matching the modern distribution of walnut. Hindcasting explicitly correlates climatic factors with species distributions and complements the genetic analysis in locating Pleistocene refugia. Our LGM hindcasts using data from the CCSM and MIROC models suggested disjunct distributions of walnut populations restricted to Transcaucasia, Central and South Asian regions extending into southwestern Tibet, northeastern India, Himalayan region of Sikkim and Bhutan, and southeastern China. CCSM and MIROC projections overlapped, but MIROC projected a significant presence of walnut in the Balkan Peninsula during the LGM (Fig 4). In contrast, population genetic analysis of the modern walnut distribution suggested a much narrower area in northern Pakistan and the surrounding areas of Afghanistan, northwestern India and southern Tajikistan, as a plausible hotspot of diversity where walnut may have survived glaciations (Fig 4). Paleo-projections of walnut distributions correspond to pollen finds reported from Ljubljana in Slovenia [114] and Staro-Orjachovo near Varna on the Black Sea coast [115], suggesting walnut occurred in the Balkan region during the Eemian interglacial, but vanished completely during the LGM [25], as projected by the hindcast with the LGM-CCSM simulated climatic data (Fig 4). During the LIG and later, walnut still occurred in the Ghab Valley in Syria [116] as shown in both LGM projections (Fig 4). The Colchis region is regarded as a glacial refugium for thermophilous plants of the Neogene flora [77, 117], and our ENM results agree with the previous report that species such as *Pterocarya* and walnut survived in this region throughout the Pleistocene [82]. Hyrcanian forests stretching from the Talysh Mountains in southeastern Azerbaijan along the southern shores of the Caspian to Golestan National Park in Iran have been an important refugia for temperate broad-leaved trees including walnut during the Quaternary glaciations [118–120] confirming our LGM and LIG projections. Climatic conditions around the Black Sea and the Caspian Sea were favorable for walnut during the last glacial period [121]. The LIG predictions suggest that walnut probably had an extended low rate of occurrence in southern and western Europe from the Iberian Peninsula through southern France, the Italian Peninsula, Adriatic Coastal regions, Greece, the Caucasus, southern Black Sea regions of Turkey to southern Russia, western Kazakhstan, East Asia, and scattered distribution in SW Asia. Palynological evidence confirms the occurrence of walnut in many of these areas during the Quaternary period. The LIG climatic predictions in higher latitudes suggest small pockets of marginal climate for a low rate of occurrence of walnut in the UK, Germany, and Sweden. However, *Juglans* pollen found in two peat bogs in Kashmir between 17,000 and 10,000 cal yr BP onward supports the results of population genetic analysis of modern walnut distribution.

## Conclusion

The paleoclimatic predictions show that the distribution of walnut was affected by Quaternary climatic fluctuations with population contractions and fragmentation. The LGM-CCSM and LGM-MIROC models suggested broad areas of the Caucasus, SW Asia including northeastern Afghanistan, Northern Pakistan, northeastern India and Central Asian Republic of Tajikistan as favorable climatic regions where walnut probably survived in multiple refugia. The LIG prediction suggested that walnut perhaps had expanded distribution in southern Europe from the Iberian Peninsula through southern France, Italian Peninsula, Adriatic Coastal regions, Greece, the Caucasus, southern Black Sea regions of Turkey up on to southern Russia, western Kazakhstan, East Asia and scattered distribution in SW Asia. Palynological evidence confirms occurrence of walnut in many of these areas during the Quaternary period. However, a cautionary note on paleoreconstructions is that they only predict the potential climatic niche suitable for species and may not confirm actual existence of refugia.

Population genetic analysis of walnut representing the modern distributional range suggested a general area of northern Pakistan and surrounding areas including northeastern Afghanistan, southern Tajikistan and northwestern India as the possible hotspot of diversity where walnut probably survived the last ice age. The genetic analysis also indicated that walnut probably spread into neighboring Central Asia, the Caucasus, West Asia and eventually Eastern Europe during the Roman period as confirmed by fossil pollen evidence. Overall, the findings suggest that walnut possibly survived the last glaciations in several refugia across a wide geographic area between 30 and 45 degrees north latitude. However, humans have played a significant role in the recent history and modern distribution of walnut.

## Supporting information

S1 Table Walnut germplasm used in the study. (XLSX)

S2 Table Multilocus walnut genotypes data for 19 microsatellite loci. (XLSX)

S3 Table Walnut occurrence data and coordinates. Walnut occurrence data with geographic coordinates sourced from the Genetic Resources Information Network (GRIN, USDA-ARS; http://npgsweb.ars-grin.gov/gringlobal/search.aspx) and Global Biodiversity Information Facility (GBIF; http://www.gbif.org).
(XLSX)

S4 Table Locus-wise private alleles in different geographic groups of walnut. (XLSX)

S5 Table Results of model tuning for the unfiltered, filtered at 10 km, and filtered at 25 km walnut occurrence data sets. Rows in bold shows the model setting with ΔAIC_c_ = 0.
(XLSX)

S1 Fig Model tuning for walnut data sets. Model tuning results for three different walnut data sets: (A) unfiltered with 237 occurrence points and two filtered data sets rarified at (B) 10 and (C) 25 km geographic resolutions with 137 and 112 occurrence points, respectively. Evaluation metrics generated from MaxEnt models with six different settings for feature Class: Linear (L); Linear and Quadratic (LQ); Hinge (H), Linear, Quadratic, and Hinge (LQH); Linear, Quadratic, Hinge, and Product (LQHP); and Linear, Quadratic, Hinge, Product, and Threshold (LQHPT) and regularization multipliers ranging from 1 to 5 with increments of 0.5.
(TIFF)

S2 Fig Unfiltered and filtered (25 km) ecological niche modeling of walnut distributions. AIC_c_-selected model prediction of occurrences of walnut for current, last glacial maximum (LGM; 21–18 kyr BP), and last interglacial (LIG; 130–107 kyr BP) climatic conditions for unfiltered data set with 237 occurrence points and filtered at 25 km spatial resolutions with 112 occurrence points, respectively (refer to Table 6 for feature class and regularization multiplier settings).
(TIF)
