# Supplementary figures and images for: Genetic and ecological insights into glacial refugia of walnut (Juglans regia L.)
Source: PLoS One. 2017 Oct 12;12(10):e0185974. doi: 10.1371/journal.pone.0185974 (PMC5638312; doi:10.1371/journal.pone.0185974)

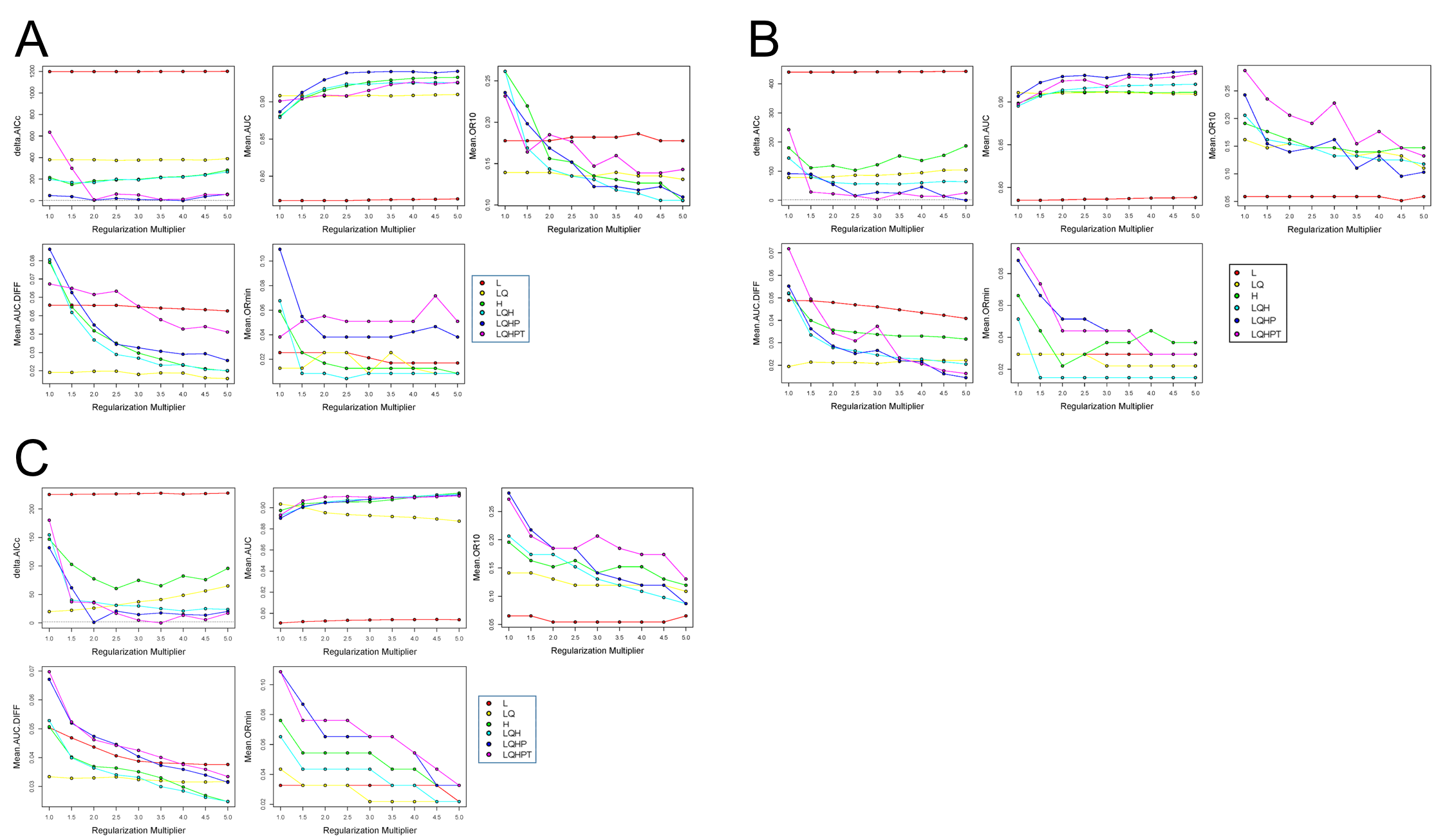

Supplement: S1 Fig — Model tuning results for three different walnut data sets: (A) unfiltered with 237 occurrence points and two filtered data sets rarified at (B) 10 and (C) 25 km geographic resolutions with 137 and 112 occurrence points, respectively. Evaluation metrics generated from MaxEnt models with six different settings for feature Class: Linear (L); Linear and Quadratic (LQ); Hinge (H), Linear, Quadratic, and Hinge (LQH); Linear, Quadratic, Hinge, and Product (LQHP); and Linear, Quadratic, Hinge, Product, and Threshold (LQHPT) and regularization multipliers ranging from 1 to 5 with increments of 0.5. (TIFF) [file pone.0185974.s006.tiff]

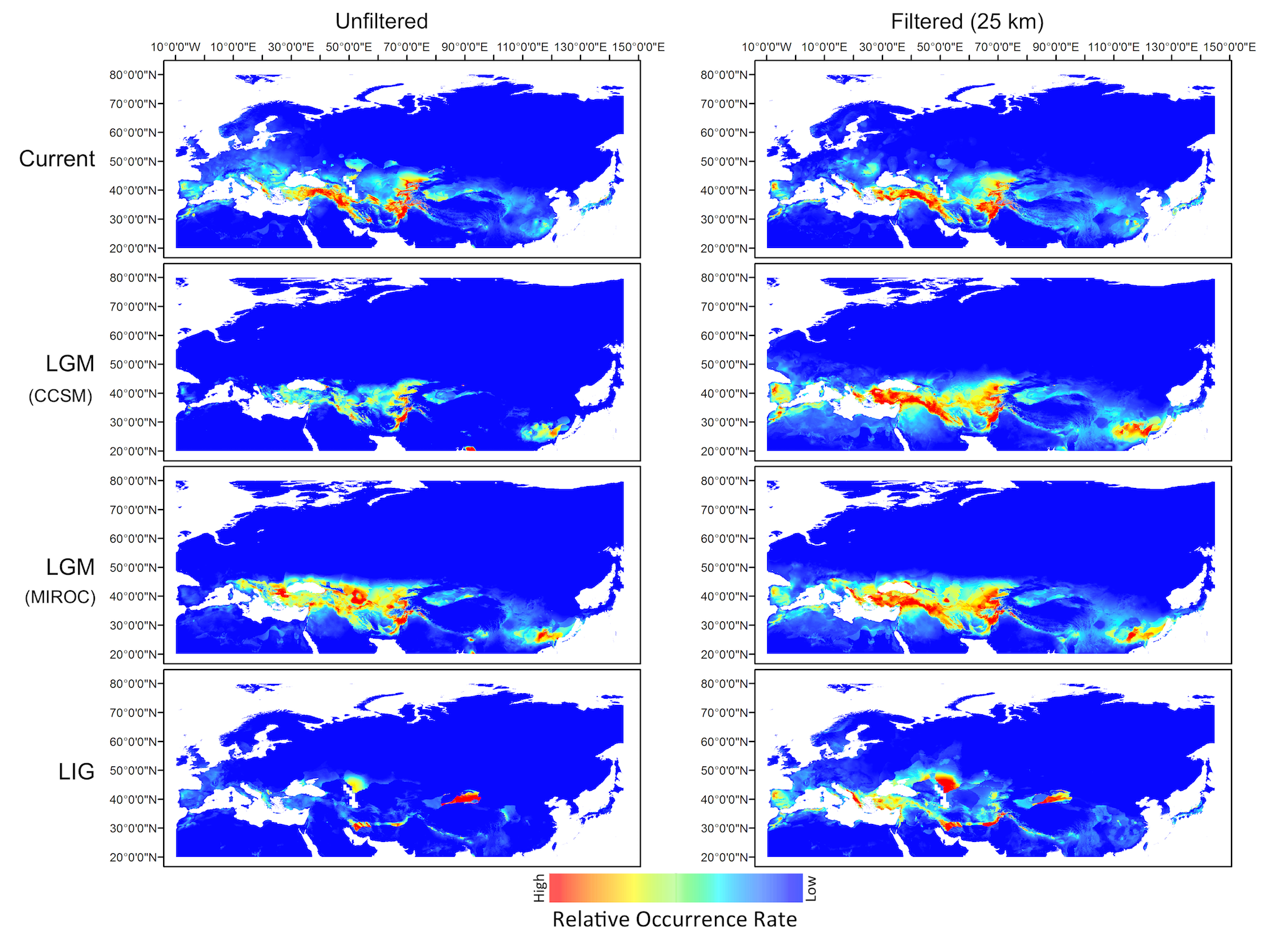

Supplement: S2 Fig — AICc-selected model prediction of occurrences of walnut for current, last glacial maximum (LGM; 21–18 kyr BP), and last interglacial (LIG; 130–107 kyr BP) climatic conditions for unfiltered data set with 237 occurrence points and filtered at 25 km spatial resolutions with 112 occurrence points, respectively (refer to Table 6 for feature class and regularization multiplier settings). (TIF) [file pone.0185974.s007.tif]
